# Monoclonal Antibody a Promising Treatment for Depression

**Published:** 2019

**Authors:** Shahin Akhondzadeh

**Affiliations:** Psychiatric Research Center, Roozbeh Hospital, Tehran University of Medical Sciences, Tehran, Iran

Major depressive disorder (MDD) is one of the prominent causes of disability in the world, affecting 15–20% of people over the period of a lifetime. Patients commonly experience continuous remaining symptoms, functional impairment, and diminished health 1.

Treatment of MDD is still far from optimal and drug resistance in MDD is still considered a serious clinical challenge. Only about one-third of patients completely respond to their first antidepressant medication, first with an approximate lag time of 2 months. This notable lag time for onset of therapeutic efficacy is associated with significant morbidity and suicidal risk. As a result, there is a widely accepted need for fast acting antidepressants. Another downside of the currently available treatments is their side effects which are documented in large proportion of patients 1,2.

Patients who do not respond to their first-line medication are generally treated by either switching to another treatment or with augmentation therapy to achieve favorable response. Combination therapy from start of treatment has been suggested recently to gain quicker and better response and remission rates, however, not all studies have supported this opinion. Most MDD pathophysiology etiological theories used to focus on brain modulatory monoamine systems (dopamine, serotonin and norepinephrine) 3,4. A more recent line of evidence points to glutamate, the brain’s principal excitatory neurotransmitter, as playing a role in MDD’s pathophysiology. Additionally, glutamate dysregulation is known to cause impairments in structural plasticity and cellular resilience, which seems to be implicated in mood disorders as well. It is therefore reasonable to hypothesize that medications which reduce glutamatergic tone may be able to play a role in treatment of depression 5.

Depression is demonstrated to be accompanied with parallel increases in the immuno-inflammatory biomarkers including significantly higher levels of pro-inflammatory cytokines interleukin (IL)-1, IL-6, tumor necrosis factor (TNF)-α, and C-reactive protein (CRP) in depressed patients compared to normal individuals. The dynamic interaction between pro-inflammatory cytokines, prostaglandin (PG)-E2 synthesis and depression has led to suggestions that anti-inflammatory agents could be useful for treatment of depression 6,7. Celecoxib, a nonsteroidal anti-inflammatory drug that acts *via* the selective inhibition of cyclooxygenase (COX)-2, has shown promising outcomes in several psychiatric diorders, including autistic disorder, schizophrenia, and depression. Antidepressive effects of celecoxib is suggested to be largely attributed to its inhibitory effect on the levels of pro-inflammatory cytokines. In line with this idea, baseline levels of serum IL-6 is demonstrated to be significantly correlated with the Hamilton Depression Rating Scale (HDRS) score. In the same article, adjunctive therapy with celecoxib, as an antidepressant, resulted in significant decreases in both serum IL-6 levels and HDRS scores. Although most of the previous research has focused on the antidepressant effects of celecoxib as an add-on treatment, few reports have investigated the safety and efficacy of celecoxib as a monotherapy 8–10.

In clinical trials, two new classes of anti-inflammatory drugs-anti-cytokine monoclonal antibodies and cytokine inhibitors have been shown to reduce inflammation in a range of autoimmune diseases, and these drugs have already started to be administered to patients who do not respond to standard treatments 11,12.
